# Circulating Cytokine Levels as Markers of Inflammation in Philadelphia Negative Myeloproliferative Neoplasms: Diagnostic and Prognostic Interest

**DOI:** 10.1155/2015/670580

**Published:** 2015-10-07

**Authors:** Julie Mondet, Kais Hussein, Pascal Mossuz

**Affiliations:** ^1^UF de Pathologie Moléculaire, Département d'Anatomie et Cytologie Pathologiques, CHU Grenoble, 38043 Grenoble Cedex 09, France; ^2^Equipe TheRex Laboratoire TIMC-IMAG-UMR5525 CNRS, Université Grenoble Alpes, France; ^3^Institute of Pathology, Hannover Medical School, Carl-Neuberg-Strasse 1, 30625 Hannover, Germany; ^4^Laboratoire d'Hématologie Cellulaire, Institut de Biologie et Pathologie, CHU Grenoble, 38043 Grenoble Cedex 09, France

## Abstract

Cytokines are well known mediators of numerous physiological and pathological processes. They contribute to the regulation of normal hematopoiesis but increasing data suggest that they also have a clinical impact in some hematopoietic malignancies. In particular, there is evidence that cytokines are implicated in the functional symptoms of Philadelphia negative myeloproliferative neoplasms (Ph− MPNs), suggesting that evaluation of circulating levels of cytokines could be of clinical interest for the characterization of patients at the time of diagnosis and for disease prognosis. In this review, we present the current knowledge on alteration of circulating cytokine profiles in MPNs and their role in myelofibrosis pathogenesis. Phenotypic correlation, prognostic value of cytokines, and impact of JAK inhibitors are also discussed.

## 1. Cytokine Networks in Myeloproliferative Neoplasms

Cytokines are known to play essential roles in hematopoiesis such as the regulation of differentiation and production of progenitor cells and mature blood cells [1]. The knowledge of cytokine function has not only contributed to the development of supportive therapies (i.e., Erythropoietin (EPO)), but dysregulation of cytokines also argues in the diagnosis of some hematopoietic disorders. For example, one of the minor criteria of polycythemia vera (PV) according to WHO 2008 classification is the subnormal serum EPO level [2]. Recently, clinical trials with Janus kinase (JAK) inhibitors have confirmed the presence of aberrant production of inflammatory cytokines and highlighted their role in the pathophysiology of Philadelphia negative myeloproliferative neoplasms (Ph− MPNs). Indeed, clinical impact of JAK inhibitors on the functional symptoms and splenomegaly in patients were concomitant with a significant effect on the plasma levels of many cytokines [3, 4].

The first experimental data that showed elevations of serum and/or plasma cytokines in Ph− MPN date back to more than 15 years. In the 90s, changes in serum levels of interleukin (IL) such as IL6 [5, 6], IL2 and its soluble receptor [7], and of tumor necrosis factor (TNF*α*) [8] were already reported associated with disturbances of blood cell counts. The study of Hermouet et al. [9] in 2002 has expanded this panel, showing elevated serum levels of IL8 and IL11 in patients with PV, compared to healthy subjects. Elevated serum concentrations of IL11 and IL8 were observed in 30% and 100%, respectively, of the PV but not in controls. This high concentration of these two cytokines was also observed in the bone marrow plasma in 48% and 100% of PV patients, respectively, concerning IL11 and IL8. The authors have also shown that the stimulation of stromal cells with IL1*β* induced an increase in the production of these two cytokines, suggesting that bone marrow stromal cells regulate IL11 and IL8 production. This study also described an elevation of IL8 both in sera and in bone marrow plasma among patients classified as idiopathic erythrocytosis (in the absence of endogenous erythroid colonies).

In 2005, Panteli et al. measured the serum levels of IL1*α*, IL1*β*, IL2, IL6, soluble IL2 receptor alpha (sIL2-Ra), and Thrombopoietin (TPO) in 25 primary myelofibrosis (PMF), 40 Essential Thrombocytemia (ET), and 8 PV in comparison with a group of 27 healthy subjects and a subgroup of 10 chronic myeloid leukemia (CML) patients [6]. The interest issue of this study was to show that all Ph− MPNs MPNs (PMF, ET, and PV) had significant increased serum levels of IL2 and its soluble receptor, compared to healthy subjects. The CML patients showed the same increases compared to the healthy subjects, but with significantly lower values than PMF. Similarly, PV and ET patients had significantly lower levels of IL2 compared to PMF ones. Overall, PMF patients displayed a gain of all cytokines measured in this study, with the exception of IL1*α* and IL1*β*, compared to healthy subjects and CML, PV, and ET patients. The profiles of ET and PV patients were relatively similar with no significant difference reported between these 2 subgroups, although the rate of IL2 and its receptor were higher in PV (but not significant). Concerning all the patients evaluated (Ph− MPN and CML), the authors did not find any significant increase of IL1*α* nor IL1*β* compared to healthy subjects.

Regarding TPO results, the authors found a significant increase in TPO serum compared to controls only for patients with PMF. ET and PV patients, despite moderately higher median levels, had no significant overexpression of TPO (versus controls), although high levels of TPO in ET have previously been reported [10, 11]. No difference between PV and ET could be demonstrated in this study. The moderate increase of TPO levels must be interpreted in view of the decreasing rates of EPO reported in several studies, in correlation with EPO independent growth of hematopoietic progenitors in MPNs. In particular, a multicenter study on a cohort of 116 PV reported a significant reduction in rates of EPO in 85% of patients compared to secondary polycythemia, confirming the interest of the diagnostic assessment of serum EPO in PV [12]. In the study of Panteli et al. [6], the observed changes do not suggest that the assay of TPO can serve as a diagnostic marker of ET. Indeed, increasing levels of TPO were not correlated to platelet count or bone marrow megakaryocyte to clumping.

In 2011, Tefferi et al. [13], using a multiplex assay using magnetic nanobeads coupled with flow cytometry, have assessed plasma levels of 30 cytokines including several growth factors such as granulocyte colony-stimulating factor (G-CSF), vascular endothelial growth factor (VEGF), and hepatocyte growth factor (HGF), in a cohort of 127 patients with PMF. The assay was compared to a control population comprising 35 healthy subjects. Firstly, this study confirmed the wide deregulation of cytokine expression described in PMF patients. In fact, 20 of the 30 cytokines tested in plasma showed significant differences, compared to healthy subjects. The authors approved the previously described increases of IL2, IL6, and IL8 but also found significant increases in IL10, IL12, IL13, IL15, TNF*α*, and interferon alpha (INF*α*). Contrary to previous results [6], the authors found elevated levels of IL1*α* and IL1*β*. This difference is probably due to the different techniques/antibodies used (conventional ELISA versus multiplex assay). In this inflammatory profile, additional deregulations of hematopoietic growth factors such as G-CSF, HGF, and VEGF were observed. Of the 127 patients included in this study, 90 patients had a blood sample taken at diagnosis before any treatment, showing that inflammatory conditions characterized by a cytokine overproduction play an integral part in the disease.

Using the same technology, Vaidya et al. were able to study the cytokine profiles of another cohort of 65 PV compared to the results obtained in their cohort of 127 PMF and 35 controls [14]. In this study, they showed that several plasma cytokines were abnormally expressed in PV compared to normal controls, but PV patients presented a different pattern to PMF patients. Compared to normal controls, PV patients demonstrated significantly higher levels of IL1RA, IL5, IL6, IL7, IL8, IL12, IL13, IFN*γ*, granulocyte-macrophage colony-stimulating factor (GM-CSF), macrophage inflammatory protein 1*α* and 1*β* (MIP-1*α* and MIP-1*β*), HGF, IFN*γ* inducible protein 10 (IP-10), monokine induced by IFN*γ* (MIG), monocyte chemotactic protein-1 (MCP-1), and VEGF. Conversely, levels of epidermal growth factor (EGF) and regulated on activation normal T-cell expressed and secreted (RANTES) were lower in PV compared to normal controls. Differences between PV and PMF were numerous. Levels of the following cytokines were significantly higher in PMF compared to PV: IL-1*β*, IL1RA, IL-2R, EGF, IL10, basic fibroblast growth factor (b-FGF), IL12, IFN*α*, and RANTES. In contrast, levels of IL7, IFN*γ*, GM-CSF, MIP-1*α*, IP-10, MIG, and VEGF were significantly higher in PV compared to PMF.

Using the same multiplex assay technology, these results were improved by Pourcelot et al., who studied the plasma concentrations of 13 cytokines in the plasma of 17 PV and added data on 21 ET [15]. This study firstly permitted to highlight a significant elevation of these 13 cytokines in PV and in ET. As in the study of Vaidya et al. [14], the authors found previously reported increases in IL6, IL8, IL12, IFN*γ*, GM-CSF, HGF, and VEGF in PV. Moreover, the study of Pourcelot et al. found a significant increase in plasma levels of IL4, IL10, MCP-1, TNF*α* (not significant in the study of Vaidya et al.), and platelet derived growth factor (PDGF-BB) (not determined in the study of Vaidya et al.). Interestingly, the authors showed that PV and ET patients differ by their plasma cytokine profiles. ET patients had higher levels of IL4, IL8, GM-CSF, IFN*γ*, MCP-1, PDGF, and VEGF compared to PV. It is interesting to note that cytokines evaluated in this study were higher in ET patients than in PV.

All these studies confirmed the existence of an inflammatory context associated with MPNs; Gangemi et al. [16] have focused on the evaluation of IL22, IL23, and IL10 circulating cytokines which are considered as markers for the activation of T helper lymphocytes. This study showed a significant elevation of IL23 in PV patients compared to controls. However, ET patients did not show any changes in these 3 cytokines compared with controls and no difference between PV and ET could be demonstrated.

**Figure 1:**
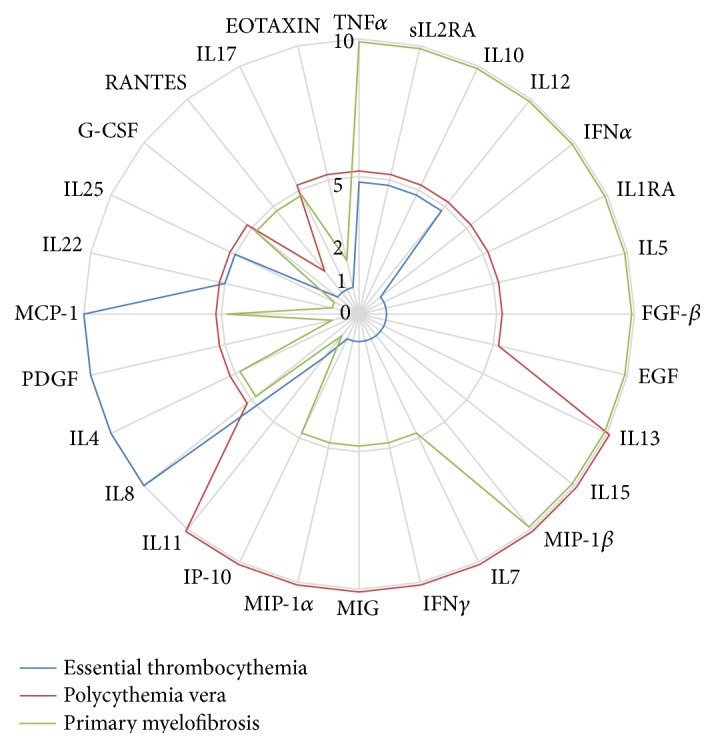
Radar graph of relative cytokine expression profiles according to MPN subtypes. Data were analysed from 6 studies [6, 13–16, 45]. For each myeloproliferative neoplasm, arbitrary scores were attributed to different cytokines according to their relative variations: 10 corresponds to an overexpression of cytokines compared to one or both MPNs; 2 corresponds to underexpression compared to one or both MPNs; 1 was attributed where no data was found in literature; 5 represents intermediary cytokine level. In cases of discordances between several studies, data are not added in this graph.

From all of these studies, several comments emerge: (i) Ph− MPNs (PV, ET, and PMF) are all characterized by a significant change in the cytokine production objectified by increased plasma levels of many inflammatory cytokines (i.e., IL1, IL2, IL6, IL8, IL12, TNF*α*, and IFN*γ*), several growth factors (e.g., GM-CSF, G-CSF, HGF, PDGF, and EGF), and angiogenic factors (i.e., VEGF) (Table 1); (ii) deregulations also concern anti-inflammatory cytokines such as IL4 and IL10; (iii) there are differences in levels and cytokine profiles among MPNs but no particular continuum between these diseases could be objectified (Figure 1). Some cytokines are overexpressed in PMF versus PV (i.e., IL1*β*, IL1RA, IL2-Ra, EGF, and IL10). Conversely, some are overexpressed in PV versus PMF (i.e., IL7, IFN*γ*, GM-CSF, MIP-1*α*, IP-10, and MIG) and, finally, ET also have higher rates than PV concerning IL6, IL8, IL12, IFN*γ*, GM-CSF, and HGF; (iv) on a technical level, in the absence of standardization of methods between different studies, it is difficult to compare these results to each other; (v) the investigation of a large cohort of MFP, PV, and ET using the same technology would clarify these differences and allows to better define the existence of specific profile of each disease; (vi) interpretation of cytokine levels should take into account other factors that may limit the ability to use cytokines in everyday practice. For example, modifications of the immune system occur with age. Consequently, in healthy patients, age was shown to increase the measurement of IL6 and interferon-gamma inducible chemokines (MIG and IP-10) and conversely to decrease IL2, EGF, and EGFR measurements [17, 18].

## 2. Megakaryocytic and Granulocytic Cytokine Production in Myelofibrosis

In the normal bone marrow, the stroma cells comprise fibrocytes/fibroblasts, endothelial cells, osteocytes/osteoblasts as well as osteoclasts. Fibrosis is the result of collagen production by fibroblasts and its deposition in the extracellular space and in parallel scar tissue formation [19]. In general, megakaryocytic clustering can be regarded as surrogate for increased megakaryocytic proliferation. In MPNs, clonal (mutated) neutrophilic or erythroid cells are morphologically indistinguishable from their polyclonal counterparts but the increase in bone marrow granulocytic progenitors and neutrophils is the morphological surrogate for aberrantly increased proliferation. Erythropoiesis is usually not increased in PMF and patients can have normal hemoglobin values or anemia [20]. The shape of the megakaryocytic nuclei and the emphasis of granulocytic proliferation are matters of dogmatic debate, in particular cloud-like (PMF) versus staghorn-like (ET) megakaryocytic nuclei [21–23].

The fundamental question is, why do fibroblasts start to produce more fibers and why are these fibers not degraded? All bone marrow cells (hematopoietic and nonhematopoietic) communicate with each other via direct cell-cell contact and via cytokine-receptor signaling. In his editorial article “Some Speculations on the Myeloproliferative Syndromes,” William Dameshek raised the hypothesis that hormonal signaling factors (“myelostimulatory factors”) may lead to these myeloid diseases [24]. Nowadays, although we have found clonal markers, aberrant expression of cell signaling molecules, and regulatory microRNAs [25–27], we still speculate on the causes of these diseases and in particular why progressive and prognostic adverse myelofibrosis develops in these patients.

**Figure 2:**
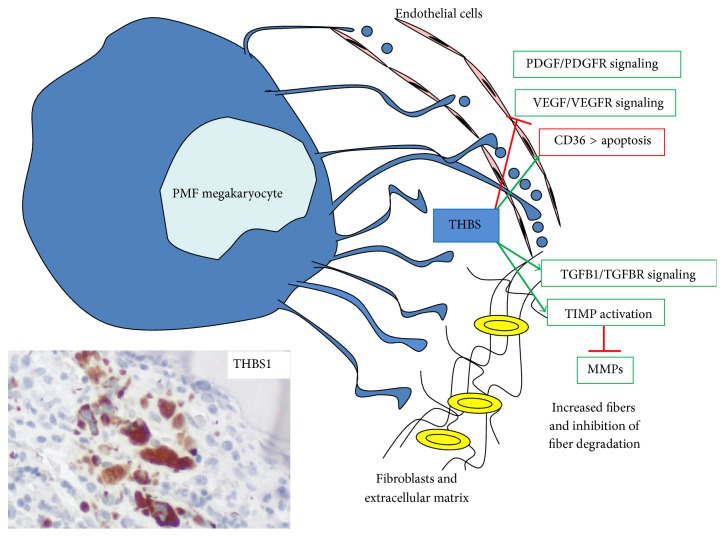
Role of THBS secreted by PMF megakaryocytes in angiogenesis and myelofibrosis development. Megakaryocytic THBS can lead to fibrosis via activation of TGF*β*1 signaling and TIMPs but simultaneously inhibits angiogenesis via its receptor CD36 and inhibition of VEGF signaling. Nevertheless, myelofibrosis is associated with increased vascularization which could be mediated by PDGF signaling. The immunohistochemical image shows strong THBS expression in clustered PMF megakaryocytes and in small proplatelet depositions* in situ*.

The fact that prefibrotic PMF has similar megakaryocytic atypia as fibrotic-stage PMF and that megakaryoblastic acute myeloid leukemia (AML) is frequently associated with fibrosis make it likely that megakaryocytes could be the neoplastic cell subtype which predominantly forces fibroblasts to produce fibers. In contrast to ET megakaryocytes, PMF megakaryocytes form a more dense net of proplatelets within the bone marrow [28]. Therefore it is possible that increased proplatelet depositions lead to increased intramedullar cytokine release. Two central fibrosis-related cytokine/receptor pairs in MPNs are PDGF and its receptor (PDGFRA) and transforming growth factor beta 1 (TGF*β*1) and the TGF type II receptor (TGFBR2) [29–31]. However, the link between the production of PDGF and TGF*β*1 and MPN-associated mutations remains unclear. The* JAK2V617F* mutation does not directly result in fibrosis although it was observed that, in PMF, the mutant allele frequency is high [32]. In fact,* JAK2V617F* is mutated in 50–60% of PMF (including MF0-3) as well as 50–60% of ET, but in almost all cases of PV [25, 32].* JAK2V617F*, through its association with PDGFRA and TGFBR2, may contribute to enhance their signaling, mimicking the action of their ligands. Currently, there is no known mutation which directly leads to myelofibrosis. It is more likely a progressive and long-lasting shift of the cytokine microenvironment towards fibrosis rather than one single genetic trigger. Several matrix modulating factors are increased, in particular thrombospondins (THBS) 1 and 2 and matrix metalloproteinases (MMPs) such as MMP14 which are produced by megakaryocytes (Figure 2) [33, 34]. MMPs degrade fibers while tissue inhibitors of metalloproteinase (TIMP) contribute to fiber accumulation but, in the most advanced stage PMF, TIMPs are not increased [34]. Therefore, increased expression of MMP14 could reflect a higher turnover of the extracellular matrix. Profibrogenic and simultaneously antiangiogenic THBS are matricellular factors which are not involved in structuring the extracellular matrix, but which regulate other factors of the extracellular matrix [35].

Fibrosis is not an isolated change but is usually accompanied by increased vascularization. It is thought that angiogenesis plays a critical role in the pathogenesis of PMF. Increased THBS acts ineffectively against exaggerated angiogenesis (e.g., mediated by increased PDGFRA levels) [29, 36]. Data from bone marrow histopathology suggest an increase in the microvessel density (MVD) and VEGF. Boveri et al. reported that PMF patients were characterized by a significant increase of MVD, particularly microvessels assessed by CD105 expression, compared to PV, ET, and controls [37]. Interestingly, they showed that prefibrotic PMF could be differentiated from ET by MVD. Similarly, post-PV and post-ET myelofibrosis harbored significantly higher numbers of microvessels compared to PV and ET, respectively. In parallel to the increasing density of microvessels, a significant increase expression of VEGF has been observed in PMF patients compared to ET, PV, and MDS/MPN [38]. Gianelli et al. confirmed high levels of VEGF expression in PMF compared to ET or controls but failed to objectify differences between PMF and PV [39]. Expression of VEGF receptor (VEGFR-1 and VEGFR-2) seems less specific. Both receptors were weakly expressed, mainly in megakaryopoietic and erythropoietic progenitors, with heterogeneous intensity [38]. MVD have been shown to correlate with a high* JAK2V617F* allele burden (≥55%) in mutated patients suggesting that angiogenesis may be influenced by allele burden in these patients, keeping in mind that about half of PMF are JAK2 wild type which clearly indicate that other factors (yet unknown mutations or aberrant cytokine expression) mediate microvessel proliferation.

## 3. Phenotypic Correlation and Prognostic Value of Circulating Levels of Cytokines

### 3.1. Correlation with Blood Cell Counts

One of the first issues is the existence of a correlation between the level of circulating cytokines and the intensity of hematopoietic production and/or the existence of specific cytokine overproduction correlated to a cell type overproduction. The study of Tefferi et al. [13] showed, in PMF, the existence of a correlation between the rate of sIL2-Ra, HGF and IP-10 (only sIL2-Ra in multivariate analysis), and leukocytosis. In PV, correlation of IL-1*β*, IL2, IL7, b-FGF, and HGF with leukocytosis has been described [14]. More precisely, Pourcelot et al. reported a correlation between IL6, TNF*α*, and the number of lymphocytes in PV, and a correlation between HGF, IL6, IL12, GM-CSF, and VEGF with the numbers of neutrophils in ET [15]. Regarding erythrocyte production, significant correlations were reported in PV between IL12 and hematocrit (Ht) [14], TNF*α*, and Ht [15] and between IL4 or MCP-1 and hemoglobin (Hb) [15]. Moreover, Pourcelot et al. found in ET a correlation between PDGF-BB and red cell counts. These results suggest a relative specificity of the plasma levels of some cytokines with the deregulation of the red cell mass. Regarding the platelet count, no cytokine plasma levels were correlated with platelet count in ET patients in the literature. However, Vaidya et al. in 2012 reported a significant reduction of INF*α* and *γ* in patients with a platelet number greater than 450, and a significant increase of IL6, capable of regulating the platelet count, has been reported for ET patients in several studies.

The difficulty in interpretation of these data is related to the fact that the mechanisms of cytokine production can be multiple (bone marrow stroma cells, tumor cells, and extra-hematopoietic cells) and the plasma cytokine changes may also be linked to chronic inflammation associated with MPNs and therefore not only reflect myeloproliferation.

### 3.2. Correlation with* JAK2V617F* Status

Surprisingly, there are few data on cytokine plasma levels correlated to* JAK2V617F *status. In their study on PMF, Tefferi et al. found a correlation in multivariate analysis between the presence of the mutation and IL1RA, IP-10, and IL2-Ra rates [13]. The study of Pourcelot et al. showed a correlation between the presence of the mutation and the plasma concentration of TNF*α* and PDGF-BB in PV and ET, respectively [15].

The impact of the* JAK2V617F *mutation on cytokine secretion seems to be restricted, suggesting that the regulation of cytokine production is done by both phenomena* JAK2V617F*-driven and not driven. On the other hand, cytokines which appear* JAK2V617F*-driven differ between PMF, PV, and ET. This suggests that there is a difference in the cytokine impact of the mutation according to the pathology or conversely that these differences of cytokine profiles could contribute to the phenotypic differences within the JAK2 mutated MPN. Indeed, when comparing* JAK2V617F* mutated ET and PV patients, significant differences in the expression of some cytokines such as IL4, IL8, IFN*γ*, and PDGF-BB have been demonstrated [15]. To summarize, IL4 rates were also correlated with Ht in this study. Differences could be related to the intensity of the allelic load. For example, TNF*α* levels were reported previously to be correlated with* JAK2V67F* allelic burden. This may explain why in PV patients who express the highest levels of JAK2 mutated, a correlation between TNF*α* and the presence of the mutation was observed. In this study, TNF*α* levels were also correlated with Ht. This suggests in PV a direct link between plasma levels of TNF*α*,* JAK2V617F* allelic burden, and increased red cell mass.

The increase of PDGF in ET patients and its correlation with the presence of* JAK2V617F *mutation probably reflect the impact of the mutation on the regulation of megakaryopoiesis via TPO and the deregulation of this pathway in mutated patients. This deregulation could induce an upregulation of the synthesis of PDGF by megakaryocytes. This suggests that, in ET patients, PDGF assay may be a functional marker of* JAK2V617F* allelic load and indirectly a marker of JAK2 activation level. Thus, the plasma level of PDGF could identify ET patients for whom a JAK2 inhibitor therapy would be the most fruitful. To our knowledge, no studies have until now described any correlation between* CALR* or* MPL* mutations and circulating cytokines.

### 3.3. Correlation with the Clinical Course of Patients (Table 2)

All MPNs are capable of inducing myelofibrosis or transforming into acute leukemia. Fibrosis progression during the chronic phase of myeloid neoplasms is regarded as an indirect surrogate of the aggressiveness of the clonal disease [20]. The mechanisms which lead to myelofibrosis, primary or secondary, are still an enigma. It is likely that in bone marrow cytokines secreted by MPN cells, in particular megakaryocytes, could induce activation of fibroblasts and endothelial cells. Moreover, circulating cytokines (not originating from bone marrow) could by themselves influence the bone marrow microenvironment and thereby contribute to the development of myelofibrosis. Hence, circulating cytokines represent an interesting opportunity of simple, accessible, and easily measurable biomarkers for the evaluation of the disease at diagnosis (in addition to usual genetic and clinical markers), but also the determination of prognosis.

In Tefferi et al. [13], the follow-up of patients naive to treatment assessed the prognostic value of some of these biomarkers. In particular, the rate of IL8, IL10, IL12, and IL15 and sIL2-Ra levels were independent predictors of low survival, correlated with DIPSS categories. The prognostic value of these biomarkers was confirmed retrospectively on 127 patients, including those who received a therapeutic treatment, proving their clinical interests. Plasma levels of IL8 and sIL2-Ra allowed a prognostic classification of patients as they showed an increase of one or two of these cytokines. Patients with elevation of at least one of these markers displayed a significantly decreased survival among both treatment-naive patients and those who had already received therapy at the time of cytokine explorations. Moreover, the study of distribution of patients according to their prognosis has shown a concomitant increase in the frequency of patients with elevation of one or two cytokines and the severity of the pathology. Thus, there were more frequently patients with an increase in one of these two markers within intermediate risk groups 1 and 2 (classified according to DIPSS plus). In addition, patients with 2 elevated markers were only found in risk group 2 patients.

In PV and ET, the study of Gangemi et al. found a link between increased levels of IL2 and its soluble receptor with progression to myelofibrosis [16]. Furthermore, the prognostic value of high levels of 13 cytokines significantly associated with a lower survival in PV patients has also been reported [13]. In univariate analysis, fibrotic transformation was significantly associated with high levels of the following cytokines: IL-1*β*, IL5, IL6, IL10, IL12, IL15, IL17, and IP-10. However, in multivariate analysis, only MIP-1*β* remained significant even when age and leukocytosis were added as covariates.

The risk of transformation in acute leukemia remains very difficult to predict. The ability to predict the evolution towards this serious complication by a circulating marker would allow early therapeutic management of these patients with a very poor prognosis. Tefferi et al. showed that PMF patients who evolved into acute leukemia had elevated IL8 and sIL2-Ra levels [13]. In particular, the elevation of IL8 levels was significantly correlated with decrease in leukemia-free survival and an increase in the incidence of transformation in acute leukemia. The predictive value of IL2 rate and its soluble receptor was also highlighted in the study of Gangemi in PMF patients progressing into acute leukemia [16]. The prognostic value of plasma levels of IL8 and sIL2-Ra has been reported in other hematological tumors [40, 41] or solid cancer (head, neck, and esophagus) [42]. To our knowledge, there is no data in the literature on predictive cytokine markers of leukemic evolution concerning PV or ET.

### 3.4. Correlation with Vascular Events

Assessment of vascular risk, particularly thrombosis, is a key element in the therapeutic management of patients for prescription of cytoreductive and antithrombotic treatments. This assessment of vascular risk is important because it could cause inappropriate exposure of patients to potentially leukemogenic drugs. This assessment is still based on indirect criteria such as age, a history of stroke, or the presence of vascular risk factors. The existence of an inflammatory context in thrombotic events has long been demonstrated, suggesting the importance of the evaluation of inflammatory cytokines in the evaluation of thrombotic risk. However, little is known about the existence of cytokine dysregulation associated with thromboembolic events in MPN and their potential predictive values.

In the study of Pourcelot et al., data on vascular events were available for 32 patients. They could therefore compare cytokine profiles in patients with or without vascular complications. Comparison of both subgroups did not show significant statistical difference for age, JAK2 mutational status, and biological parameters (leukocytes, platelets, numbers of neutrophils and lymphocytes, red cells, Hb, and Ht). Except for IL12 (p70) which was increased in patients with vascular complications, there were no significant differences in other cytokine levels between patients with or without vascular complications [15]. Comparison of vascular complications within PV and ET revealed a significant difference of IL12 and GM-CSF in the PV subgroup. Both parameters were increased in PV patients without complications. No significant difference was observed concerning ET patients.

The decrease of IL12 has been previously reported in patients presenting a thrombotic event without any MPN diagnosis [43] suggesting that this cytokine is a specific marker of the occurrence of thromboembolic events independent of MPN pathogenesis. However, these results must be interpreted with caution since some thrombotic events occurred before diagnosis, and therefore the cytokine evaluation was done after the accident. This may explain the difficulty in highlighting cytokine alterations predictive of thrombotic risk. It would be necessary to assess changes in cytokine levels at multiple times, before and after thrombosis, to identify more specifically predictive biomarkers of vascular complications.

A study of Barbui et al. has focused on the interest of C-reactive protein (CRP) and pentraxin 3 (PTX3) as markers of thrombotic risk [44]. In this study of 244 ET and PV patients, a difference in prognostic value was observed between these 2 markers. High thrombosis risk patients were characterized by a significant increase (>3rd percentile) of CRP. Conversely, these patients had significantly decreased rates of PTX3. Prognostic stratification based on serum levels of these two inflammation markers has shown that patients with high CRP and low PTX3 levels had a significantly higher risk of thrombotic stroke (OR = 2.66, *P* = 0.045). In addition, the levels of these two markers were correlated with the mutational status of patients and with an allelic load greater than 50%.

## 4. Impact of JAK Inhibitors on Levels of Cytokines

In this review, we highlight the usefulness of cytokines as potent markers of prognosis. But in which way do treatments especially JAK inhibitors modify cytokines and cytokine levels? Treatment with ruxolitinib, the first JAK inhibitors approved in myelofibrosis, leads to a rapid and sustained downregulation of cytokine levels in myelofibrosis patients [3]. Another proof of the action of ruxolitinib on cytokine levels is the withdrawal syndrome consistent with cytokine storm observed after its discontinuation [46]. Independently of JAK status or of MPN subtype (myelofibrosis or PV), several cytokines were reduced after ruxolitinib treatment such as IL1Ra, IL6, IL8, TNF*α*, and bFGF [3, 4]. Only EPO and leptin were increased after ruxolitinib treatment. Reduction of IL16, IL18, VEGF, and MIP-1*β* was also reported more specifically in myelofibrosis and reduction of sIL2RA, sIL6R in PV. Moreover, a correlation between symptomatic reduction of the spleen size in myelofibrosis and reductions of IL-1ra, MIP-1*β*, IL6, and TNF*α* was observed [3]. Data reported on mouse models and on supernatants of* in vitro* cultures of mononuclear cells confirmed reductions of IL6 and TNF*α* after JAK1/2 inhibitors [47, 48]. Kleppe et al. showed that ruxolitinib treatment normalizes cytokine levels in mice transplanted with* JAK2V617F*-mutant as well as those transplanted with MPLW515L-mutant cells [49]. Beyond MPN, there is a rising interest in JAK inhibitors for other disorders such as autoimmune diseases, solid cancers, or other hematopoietic malignancies [50–52]. Reduction of cytokines was also observed in experimental models of those disorders [53, 54].

Nowadays, the only JAK inhibitor approved for the treatment of primary and secondary myelofibrosis is ruxolitinib, which inhibits not only JAK2 but also JAK1. Nevertheless, more selective JAK2 inhibitors (i.e., fedratinib, lestaurtinib, pacritinib) are in clinical development and differences between selective JAK2 inhibitors and JAK1/JAK2 inhibitors could be observed. On one hand, more selective JAK2 inhibitors appear to have a less pronounced anticytokine effect and, on the other hand, they induce a more pronounced antierythropoiesis effect [55]. For example, no consistent changes in levels of proinflammatory cytokines (IL6, IL2, IL8, and TNF*α*) relative to baseline were observed during the course of fedratinib treatment; however a rapid and durable improvement of symptoms concomitantly with an impact on* JAK2V617F* allele burden was induced [56]. Similarly,* Santos et al.* studied effects of lestaurtinib (CEP701), a selective JAK2 inhibitor, on the levels of 19 cytokines (IL-1*β*, IL-1Ra, IL2, IL6, IL8, IL9, IL10, IL12, IL13, IL15, bFGF, GM-CSF, IFN*γ*, IP-10, MIP-1*α*, MIP-1*β*, RANTES, TNF-*α*, and VEGF) [57]. In the same way, no significant change between baseline and treatment was noticed even in responders. In contrast to selective JAK2 inhibitors, momelotinib (CYT387), a JAK1/JAK2 inhibitor, normalized inflammatory cytokines in* JAK2V617F*-transduced mice [58].

Even if JAK inhibitors affect cytokine levels, recent studies suggest that cytokine regulation by JAK inhibitors is not enough by itself to fully abort this aberrant inflammatory cytokine production. Keohane et al. showed in myelofibrosis, PV, or ET patients receiving either ruxolitinib or fedratinib a significant decrease of cytokines after the first month (IFN*α*, IFN*γ*, IL10, IL2R, IL4, and IL17) but a weak rise in cytokine levels after six months [48]. This fact argues a possible therapeutic failure but also supports the interest for drug associations in MPN treatment targeting other molecular pathways implicated in inflammatory response. Moreover, the role of tumor microenvironment in hematopoietic neoplasms development is essential; not only malignant cells but also nonmalignant cells induce cytokine dysregulation [59]. Altogether, those studies suggest the interest of synergistic associations of JAK inhibitors with others drugs to normalize aberrant cytokine production in MPN.

## 5. Conclusion

The cytokine profiles of MPN patients involving deregulation of proinflammatory and anti-inflammatory cytokines as well as growth factors suggest that the impact of these deregulations is involved in hematological but also extra-hematological manifestations of these pathologies. These deregulations confirm the existence of an inflammatory reaction in MPNs that may contribute to the initiation and progression of the disease. In this context, circulating cytokine levels could be useful markers of MPNs for their characterization at diagnosis but could also be interesting in prognostic evaluation of these patients. Moreover, the impact of JAK2 inhibitors on plasma concentrations of inflammatory cytokines suggests that circulating cytokine assays could be useful to monitor therapeutic efficacy of these molecules. Long-term treatment with JAK2 inhibitors may also raise the question of patient compliance with their treatment. As a result, the significant reduction of cytokines by mechanisms of inhibition of JAK2, and most likely JAK1, could serve as an indirect marker for evaluation of therapeutic compliance in case of absence or inadequate therapeutic response.

## Figures and Tables

**Table 1:** Circulating cytokine expression in myeloproliferative neoplasms compared to healthy controls.

Types of cytokines	All MPNs	Essential thrombocythemia	Polycythemia vera	Primary myelofibrosis
Hematopoietic growth factors	↑ TPO [6, 10] NS IL6 [6, 8]	NS TPO [6] NS IL6 [6]	NS TPO [6] ↑ or NS IL6 [6, 14, 45] NS G-CSF [14] ↑ GM-CSF [14] ↑ IL5 [13, 14] ↑ IL7 [14] ↑ IL11 [9, 45]	↑ TPO [6] ↑ IL6 [6, 13] ↑ G-CSF [13]

Chemokines	ND	ND	↑ IL8 [9, 14, 45] ↑ IP-10 [14] ↑ MCP1 [14, 45] ↑ MIP1*α* [14] ↑ MIP1*β* [14] ↑ MIG [14] ↑ RANTES [14] NS EOTAXIN [14]	↑ IL8 [13] ↑ IP-10 [13] ↑ MCP1 [13] ↑ MIP1*α* [13] ↑ MIP1*β* [13] ↑ MIG [13]

Anti-inflammatory cytokines	↑ IL2 [6, 8] NS IL10 [8, 16]	↑ IL2 [6, 8, 14]	↑ or NS IL2 [6, 8, 14] ↑ IL1-Ra [14] NS IL10 [14] ↑ IL13 [14] ↑ HGF [14, 45] NS IL4 [14]	↑ IL2 [6, 8] ↑ IL1-Ra [13] ↑ IL10 [13] ↑ IL13 [13] ↑ HGF [13]

Proinflammatory cytokines	↑ sIL2-Ra [6, 8] ↑ TNF*α* [8] ↑ IL23 [16]	↑ sIL2-Ra [6, 8] NS IL23	↑ or NS sIL2-Ra [6, 8, 14] NS TNF*α* [14] ↑ IL23 [16] NS IL1*β* [14] ↑ IL12 [14] ↑ IL15 [14] ↑ IFN*γ* [14]	↑ sIL2-Ra [6, 8, 13] ↑ TNF*α* [13] ↑ IL1*β* [13] ↑ IL12 [13] ↑ IL15 [13] ↓ IFN*γ* [13]

Angiogenesis	ND	ND	↑ VEGF [14, 39] ↓ EGF [14] NS b-FGF [14]	↑ VEGF [13, 39]

Others	NS IL22 [16]	ND	NS INF*α* [14] ↑ Leptin [45]	↑ INF*α* [13] ND

This table summarizes cytokine expression possibly used as biomarkers in all myeloproliferative neoplasms (MPNs), essential thrombocythemia, polycythemia vera, and primary myelofibrosis compared to healthy donors. Because of the pleiotropic function of cytokines, they were arbitrarily classified according to proinflammatory, anti-inflammatory, hematopoietic growth factors, angiogenesis factors, chemokines, and others. References are reported in brackets. ↑ or ↓ means, respectively, increase and decrease of cytokine levels compared to healthy donors; NS means nonsignificant; ND means nondetermined.

**Table 2:** Cytokines with prognostic implications.

	Cytokines involved		
	Primary myelofibrosis	Essential thrombocythemia	Polycythemia vera

Low survival High DIPSS categories	↑ IL8 and/or sIL2Ra [13] ↑ IL10 [13] ↑ IL12 [13] ↑ IL15 [13]	ND	MIP-1*β* [13]

Progression to myelofibrosis	—	↑ IL2 [16] ↑ IL2ra [16]	↑ IL2 [16] ↑ IL2ra [16]

Leukemic transformation	↑ IL8 and/or sIL2Ra [13] ↑ IL2 and sIL2Ra [6, 8, 16] ↑ IL6 [6]	ND	ND

Vascular events	ND	ND	↑ IL12 [15] ↑ GM-CSF [15]

This table represents prognostic values of cytokines described in PV, ET, and PMF. ↑ means increase in cytokine level; ND means not described to our knowledge.

## References

[1] Majka M., Janowska-Wieczorek A., Ratajczak J. (2001). Numerous growth factors, cytokines, and chemokines are secreted by human CD34^+^ cells, myeloblasts, erythroblasts, and megakaryoblasts and regulate normal hematopoiesis in an autocrine/paracrine manner. *Blood*.

[2] Vardiman J. W., Thiele J., Arber D. A. (2009). The 2008 revision of the World Health Organization (WHO) classification of myeloid neoplasms and acute leukemia: rationale and important changes. *Blood*.

[3] Verstovsek S., Kantarjian H., Mesa R. A. (2010). Safety and efficacy of INCB018424, a JAK1 and JAK2 inhibitor, in myelofibrosis. *The New England Journal of Medicine*.

[4] Verstovsek S., Passamonti F., Rambaldi A. (2014). A phase 2 study of ruxolitinib, an oral JAK1 and JAK2 inhibitor, in patients with advanced polycythemia vera who are refractory or intolerant to hydroxyurea. *Cancer*.

[5] Hollen C. W., Henthorn J., Koziol J. A., Burstein S. A. (1991). Elevated serum interleukin-6 levels in patients with reactive thrombocytosis. *British Journal of Haematology*.

[6] Panteli K. E., Hatzimichael E. C., Bouranta P. K. (2005). Serum interleukin (IL)-1, IL-2, sIL-2Ra, IL-6 and thrombopoietin levels in patients with chronic myeloproliferative diseases. *British Journal of Haematology*.

[7] Kawatani T., Endo A., Tajima F., Ooi S., Kawasaki H. (1997). Clinical significance of serum soluble interleukin-2 receptor in chronic myeloproliferative disorders. *International Journal of Hematology*.

[8] Bourantas K. L., Hatzimichael E. C., Makis A. C. (1999). Serum beta-2-microglobulin, TNF-*α* and interleukins in myeloproliferative disorders. *European Journal of Haematology*.

[9] Hermouet S., Godard A., Pineau D. (2002). Abnormal production of interleukin (IL)-11 and IL-8 in polycythaemia vera. *Cytokine*.

[10] Griesshammer M., Hornkohl A., Nichol J. L. (1998). High levels of thrombopoietin in sera of patients with essential thrombocythemia: cause or consequence of abnormal platelet production?. *Annals of Hematology*.

[11] Karakus S., Ozcebe O. I., Haznedaroglu I. C. (2002). Circulating thrombopoietin in clonal versus reactive thrombocytosis. *Hematology*.

[12] Mossuz P., Girodon F., Donnard M. (2004). Diagnostic value of serum erythropoietin level in patients with absolute erythrocytosis. *Haematologica*.

[13] Tefferi A., Vaidya R., Caramazza D., Finke C., Lasho T., Pardanani A. (2011). Circulating interleukin (IL)-8, IL-2R, IL-12, and IL-15 levels are independently prognostic in primary myelofibrosis: a comprehensive cytokine profiling study. *Journal of Clinical Oncology*.

[14] Vaidya R., Gangat N., Jimma T. (2012). Plasma cytokines in polycythemia vera: phenotypic correlates, prognostic relevance, and comparison with myelofibrosis. *American Journal of Hematology*.

[15] Pourcelot E., Trocme C., Mondet J., Bailly S., Toussaint B., Mossuz P. (2014). Cytokine profiles in polycythemia vera and essential thrombocythemia patients: clinical implications. *Experimental Hematology*.

[16] Gangemi S., Allegra A., Pace E. (2012). Evaluation of interleukin-23 plasma levels in patients with polycythemia vera and essential thrombocythemia. *Cellular Immunology*.

[17] Shurin G. V., Yurkovetsky Z. R., Chatta G. S., Tourkova I. L., Shurin M. R., Lokshin A. E. (2007). Dynamic alteration of soluble serum biomarkers in healthy aging. *Cytokine*.

[18] Fulop T., Larbi A., Douziech N., Levesque I., Varin A., Herbein G. (2006). Cytokine receptor signalling and aging. *Mechanisms of Ageing and Development*.

[19] Sarrazy V., Billet F., Micallef L., Coulomb B., Desmoulière A. (2011). Mechanisms of pathological scarring: role of myofibroblasts and current developments. *Wound Repair and Regeneration*.

[20] Swerdlow S. H., Campo E., Harris H. L. (2008). *WHO Classification of Tumors of Haematopoietic and Lymphoid Tissues*.

[21] Wilkins B. S., Erber W. N., Bareford D. (2008). Bone marrow pathology in essential thrombocythemia: interobserver reliability and utility for identifying disease subtypes. *Blood*.

[22] Buhr T., Hebeda K., Kaloutsi V., Porwit A., Van der Walt J., Kreipe H. (2012). European Bone Marrow Working Group trial on reproducibility of World Health Organization criteria to discriminate essential thrombocythemia from prefibrotic primary myelofibrosis. *Haematologica*.

[23] Thiele J., Orazi A., Kvasnicka H. M. (2012). European Bone Marrow Working Group trial on reproducibility of World Health Organization criteria to discriminate essential thrombocythemia from prefibrotic primary myelofibrosis. Haematologica 2012;97(3):360–5—comment. *Haematologica*.

[24] Dameshek W. (1951). Some speculations on the myeloproliferative syndromes. *Blood*.

[25] James C., Ugo V., Le Couédic J.-P. (2005). A unique clonal JAK2 mutation leading to constitutive signalling causes polycythaemia vera. *Nature*.

[26] Kreipe H., Büsche G., Bock O., Hussein K. (2012). Myelofibrosis: molecular and cell biological aspects. *Fibrogenesis & Tissue Repair*.

[27] Hussein K. (2012). Pathobiology of the microRNA system. *Pathologe*.

[28] Muth M., Büsche G., Bock O., Hussein K., Kreipe H. (2010). Aberrant proplatelet formation in chronic myeloproliferative neoplasms. *Leukemia Research*.

[29] Bock O., Loch G., Büsche G., von Wasielewski R., Schlué J., Kreipe H. (2005). Aberrant expression of platelet-derived growth factor (PDGF) and PD6F receptor-*α* is associated with advanced bone marrow fibrosis in idiopathic myelofibrosis. *Haematologica*.

[30] Bock O., Loch G., Schade U., von Wasielewski R., Schlué J., Kreipe H. (2005). Aberrant expression of transforming growth factor *β*-1 (TGF*β*-1) per se does not discriminate fibrotic from non-fibrotic chronic myeloproliferative disorders. *Journal of Pathology*.

[31] Bock O., Muth M., Theophile K. (2009). Identification of new target molecules PTK2, TGFBR2 and CD9 overexpressed during advanced bone marrow remodelling in primary myelofibrosis. *British Journal of Haematology*.

[32] Hussein K., Büsche G., Schlue J., Lehmann U., Kreipe H. (2012). Myeloproliferative neoplasms: histopathological and molecular pathological diagnosis. *Pathologe*.

[33] Muth M., Engelhardt B. M., Kröger N. (2011). Thrombospondin-1 (TSP-1) in primary myelofibrosis (PMF)—a megakaryocyte-derived biomarker which largely discriminates PMF from essential thrombocythemia. *Annals of Hematology*.

[34] Bock O., Neuse J., Hussein K. (2006). Aberrant collagenase expression in chronic idiopathic myelofibrosis is related to the stage of disease but not to the JAK2 mutation status. *The American Journal of Pathology*.

[35] Chong H. C., Tan C. K., Huang R.-L., Tan N. S. (2012). Matricellular proteins: a sticky affair with cancers. *Journal of Oncology*.

[36] Fernando N. T., Koch M., Rothrock C. (2008). Tumor escape from endogenous, extracellular matrix-associated angiogenesis inhibitors by up-regulation of multiple proangiogenic factors. *Clinical Cancer Research*.

[37] Boveri E., Passamonti F., Rumi E. (2008). Bone marrow microvessel density in chronic myeloproliferative disorders: a study of 115 patients with clinicopathological and molecular correlations. *British Journal of Haematology*.

[38] Medinger M., Skoda R., Gratwohl A. (2009). Angiogenesis and vascular endothelial growth factor-/receptor expression in myeloproliferative neoplasms: correlation with clinical parameters and *JAK2-V617F* mutational status. *British Journal of Haematology*.

[39] Gianelli U., Vener C., Raviele P. R. (2007). VEGF expression correlates with microvessel density in Philadelphia chromosome-negative chronic myeloproliferative disorders. *American Journal of Clinical Pathology*.

[40] Kornblau S. M., McCue D., Singh N., Chen W., Estrov Z., Coombes K. R. (2010). Recurrent expression signatures of cytokines and chemokines are present and are independently prognostic in acute myelogenous leukemia and myelodysplasia. *Blood*.

[41] Nakase K., Kita K., Kyo T., Tsuji K., Katayama N. (2012). High serum levels of soluble interleukin-2 receptor in acute myeloid leukemia: correlation with poor prognosis and CD_4_ expression on blast cells. *Cancer Epidemiology*.

[42] Le Q.-T., Fisher R., Oliner K. S. (2012). Prognostic and predictive significance of plasma HGF and IL-8 in a phase III trial of chemoradiation with or without tirapazamine in locoregionally advanced head and neck cancer. *Clinical Cancer Research*.

[43] Reitsma P. H., Rosendaal F. R. (2004). Activation of innate immunity in patients with venous thrombosis: the Leiden Thrombophilia Study. *Journal of Thrombosis and Haemostasis*.

[44] Barbui T., Carobbio A., Finazzi G. (2011). Inflammation and thrombosis in essential thrombocythemia and polycythemia vera: different role of C-reactive protein and pentraxin 3. *Haematologica*.

[45] Boissinot M., Cleyrat C., Vilaine M., Jacques Y., Corre I., Hermouet S. (2011). Anti-inflammatory cytokines hepatocyte growth factor and interleukin-11 are over-expressed in Polycythemia vera and contribute to the growth of clonal erythroblasts independently of JAK2V617F. *Oncogene*.

[46] Tefferi A., Pardanani A. (2011). Serious adverse events during ruxolitinib treatment discontinuation in patients with myelofibrosis. *Mayo Clinic Proceedings*.

[47] Quintás-Cardama A., Vaddi K., Liu P. (2010). Preclinical characterization of the selective JAK1/2 inhibitor INCB018424: therapeutic implications for the treatment of myeloproliferative neoplasms. *Blood*.

[48] Keohane C., Kordasti S., Seidl T. (2015). JAK inhibition induces silencing of T Helper cytokine secretion and a profound reduction in T regulatory cells. *British Journal of Haematology*.

[49] Kleppe M., Kwak M., Koppikar P. (2015). JAK-STAT pathway activation in malignant and nonmalignant cells contributes to MPN pathogenesis and therapeutic response. *Cancer Discovery*.

[50] Zeiser R., Burchert A., Lengerke C. (2015). Ruxolitinib in corticosteroid-refractory graft-versus-host disease after allogeneic stem cell transplantation: a multi-center survey. *Leukemia*.

[51] Gadina M. (2013). Janus kinases: an ideal target for the treatment of autoimmune diseases. *Journal of Investigative Dermatology Symposium Proceedings*.

[52] Gu L., Talati P., Vogiatzi P. (2014). Pharmacologic suppression of JAK1/2 by JAK1/2 inhibitor AZD1480 potently inhibits IL-6-induced experimental prostate cancer metastases formation. *Molecular Cancer Therapeutics*.

[53] Liu Y., Holdbrooks A. T., De Sarno P. (2014). Therapeutic efficacy of suppressing the JAK/STAT pathway in multiple models of experimental autoimmune encephalomyelitis. *The Journal of Immunology*.

[54] Stump K. L., Lu L. D., Dobrzanski P. (2011). A highly selective, orally active inhibitor of Janus kinase 2, CEP-33779, ablates disease in two mouse models of rheumatoid arthritis. *Arthritis Research and Therapy*.

[55] Tefferi A. (2012). JAK inhibitors for myeloproliferative neoplasms: clarifying facts from myths. *Blood*.

[56] Pardanani A., Gotlib J. R., Jamieson C. (2011). Safety and efficacy of TG101348, a selective JAK2 inhibitor, in myelofibrosis. *Journal of Clinical Oncology*.

[57] Santos F. P. S., Kantarjian H. M., Jain N. (2010). Phase 2 study of CEP-701, an orally available JAK2 inhibitor, in patients with primary or post-polycythemia vera/essential thrombocythemia myelofibrosis. *Blood*.

[58] Tyner J. W., Bumm T. G., Deininger J. (2010). CYT387, a novel JAK2 inhibitor, induces hematologic responses and normalizes inflammatory cytokines in murine myeloproliferative neoplasms. *Blood*.

[59] Belver L., Ferrando A. A. (2015). Aberrant cytokine production by nonmalignant cells in the pathogenesis of myeloproliferative tumors and response to JAK inhibitor therapies. *Cancer Discovery*.

